# Demonstrating the presence of *Ehrlichia canis* DNA from different tissues of dogs with suspected subclinical ehrlichiosis

**DOI:** 10.1186/s13071-020-04363-0

**Published:** 2020-10-15

**Authors:** Carlos A. Rodríguez-Alarcón, Diana M. Beristain-Ruiz, Angélica Olivares-Muñoz, Andrés Quezada-Casasola, Federico Pérez-Casio, Jesús A. Álvarez-Martínez, Jane Tapia-Alanís, José J. Lira-Amaya, Ramón Rivera-Barreno, Orlando S. Cera-Hurtado, José A. Ibancovichi-Camarillo, Luis Soon-Gómez, Jaime R. Adame-Gallegos, Julio V. Figueroa-Millán

**Affiliations:** 1 Veterinary Science Department, Universidad Autónoma de Ciudad Juárez, Anillo Envolvente y Estocolmo s/n, Zona PRONAF, 32310 Juárez, México; 2 Veterinary and Zootechnic Faculty, Universidad Veracruzana, Miguel Ángel de Quevedo s/n esq. Yáñez, Col. Unidad Veracruzana, 91710 Veracruz, México; 3 CENID-Salud Animal e Inocuidad. INIFAP, Km. 11.5 de la Carretera Federal Cuernavaca-Cuautla, 62550 Col. Progreso Jiutepec, México; 4 Department of Veterinary Anesthesia, Analgesia and Pharmacology, Faculty of Veterinary Medicine, Universidad Autónoma del Estado de México, El Cerrillo Piedras Blancas, 50090 Toluca, México; 5 Municipal Anti-Rabies Center, Jurisdicción Sanitaria II, Servicios de Salud de Chihuahua, Calle Sevilla 4241, Colonia San Antonio, 32250 Juárez, México; 6 Faculty of Chemistry, Universidad Autónoma de Chihuahua, Campus 2, Circuito Universitario s/n, 31125 Chihuahua, México

**Keywords:** *Ehrlichia canis*, Biopsies, Spleen, Bone marrow, Liver, Lymph node

## Abstract

**Background:**

Nowadays, *Ehrlichia canis* receives increasing attention because of its great morbidity and mortality in animals. Dogs in the subclinical and chronic phases can be asymptomatic, and serological tests show cross-reactivity and fail to differentiate between current and past infections. Moreover, there could be low parasitaemia, and *E. canis* might be found only in target organs, hence causing results to be negative by polymerase chain reaction (PCR) on blood samples.

**Methods:**

We evaluated by PCR the prevalence of *E. canis* in blood, liver, spleen, lymph node and bone marrow samples of 59 recently euthanised dogs that had ticks but were clinically healthy.

**Results:**

In total, 52.55% of the blood PCRs for *E. canis* were negative, yet 61.30% yielded positive results from tissue biopsies and were as follows: 63.15% from bone marrow; 52.63% from liver; 47.36% from spleen; and 15.78% from lymph node. In addition, 33% had infection in three tissues (spleen, liver and bone marrow).

**Conclusions:**

Our results show the prevalence of *E. canis* from tissues of dogs that were negative by blood PCR. *Ehrlichia canis* DNA in tissue was 30% lower in dogs that tested negative in PCR of blood samples compared to those that were positive. However, it must be taken into account that some dogs with negative results were positive for *E. canis* in other tissues.
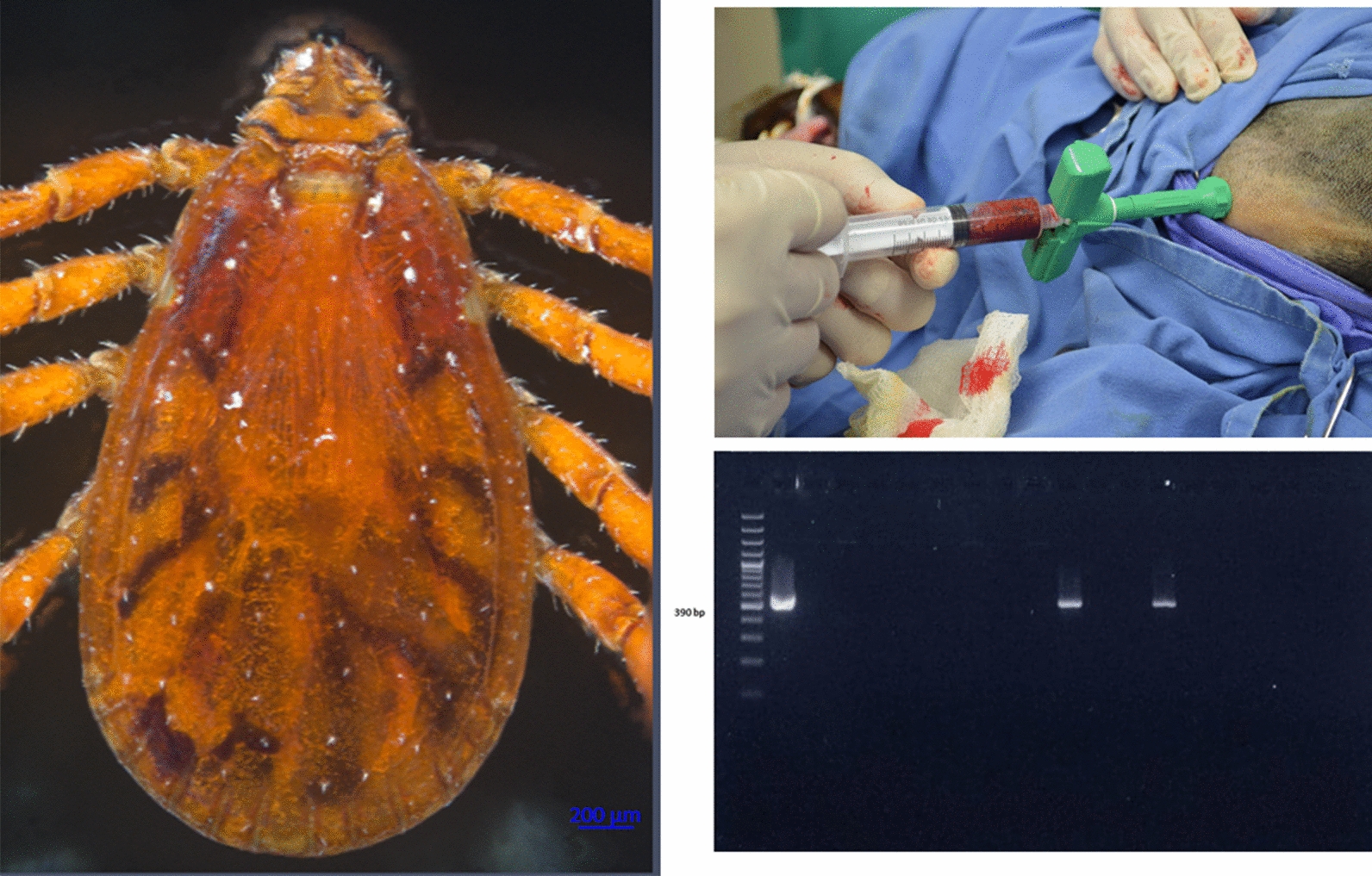

## Background

Canine monocytic ehrlichiosis (CME) is caused by *Ehrlichia canis*, an intracellular parasitic bacterium and tick-borne pathogen. Recently, this pathogen has received further attention because it has led to increasing morbidity and mortality in animals [1]. Transmission is mediated by the tick *Rhipicephalus sanguineus* (*sensu lato*), and, before infection, the bacteria replicate in monocytes and macrophages [2].

Clinical presentation of CME results in acute, chronic or subclinical phases, with several clinical manifestations. The acute phase persists for 2–4 weeks [3] and is characterised by signs in diverse systems, yet the most common are fever, weight loss, anorexia, depression, lymphadenomegaly, splenomegaly and vasculitis [4]. In addition, dogs in this phase show thrombocytopenia as the most common laboratory abnormality [5]. In the subclinical phase, dogs have persistent thrombocytopenia and leukopenia in laboratory analysis; however, during this stage, in some dogs the thrombocytopenia may be mild to non-existent [6], and they usually do not show clinical signs. The duration of this phase varies from months to years [7]. Additionally, during this phase it is common that the microorganism may not circulate in the blood but is deposited in some target organ, such as the spleen, bone marrow or liver [8–11]. Furthermore, previous research has shown that *E. canis* is widely distributed in different organs of infected dogs [8, 9, 12, 13]. Otherwise, in the chronic phase dogs show severe pancytopenia, haemorrhagic diathesis, and general debilitation [3]. Immune system deficiency, stress, co-infections, virulence strain, and geographical region are factors that influence the presentation of this phase in affected dogs [8].

In recent times, diagnosis of the disease has been challenging for practicing veterinarians [14, 15]. Identification of morulae in monocytes in a blood smear is diagnostic of the disease; however, a low frequency of morulae in buffy coat smears has previously been reported, which could be due to the low parasitaemia observed in the natural infection [7, 11, 16–21]. Besides, other more specific methods are used as diagnostics, including the immunofluorescence antibody test (IFA) and ELISA (enzyme-linked immunosorbent assay), which are both able to detect specific antibodies [21–27], as well as other molecular techniques such as the polymerase chain reaction (PCR) [1, 19, 28–36]. Presently, the Infectious Disease Group of the American College of Veterinary Internal Medicine (ACVIM) requires that dogs diagnosed with this disease show suggestive clinical signs and have positive tests, either by serology and/or by PCR [37]. A complication in the diagnosis comes about in dogs in the subclinical phase of the disease because dogs normally do not show clinical signs. Furthermore, cross-reactivity and a failure to differentiate between current and past infections with ELISA and IFA tests has been reported [25, 38, 39]. On the other hand, both in the subclinical and chronic phases, there is a possibility that parasitaemia is low in the dog [20, 26, 40, 41], as the bacteria are located in the target organs [10]. Therefore, in these cases, the dogs will be negative in a PCR blood test [10].

Presently, the presence of the DNA of *E. canis* in several tissues, such as blood, bone marrow, spleen, liver, kidney and lymph nodes has been demonstrated by PCR in experimentally infected dogs [7, 8, 11, 12, 40].

The goal of this study was to evaluate the occurrence of *E. canis* in different tissues, such as liver, spleen, lymph nodes and bone marrow, in dogs naturally infected with monocytic ehrlichiosis, assuming that a considerable percentage of dogs negative to *E. canis* by blood PCR will show positive results in biopsies of different tissues.

## Methods

An analysis of the variation in infection by *E. canis* in four tissues was carried out in two groups of dogs: positive and negative by PCR of blood samples.

### Animals

Fifty-nine dogs obtained from the municipal Anti-Rabies Centre of Juárez were used in this study. Based on the Centre’s internal regulations, animals that were not adopted 8 weeks after their arrival were euthanised. Euthanasia was performed by an overdose of sodium pentobarbital according to national and international animal welfare regulations.

In order to increase the possibility that dogs will present the subclinical phase of the disease, the inclusion criteria were that the dogs should have ticks but be clinically healthy; therefore, dogs without ticks or with signs of any disease were excluded.

### Sample collection

Whole blood samples were collected in tubes containing EDTA (Vacutainer BD^®^, Mexico City, Mexico) by cephalic venepuncture with prior administration of sodium pentobarbital. The other tissue samples were acquired by biopsies immediately after euthanasia, following the steps of surgical asepsis in order to prevent cross-contamination. In addition, with the same purpose, a change of instruments was made for the biopsy of each tissue, and particular attention was taken to avoid blood or other fluid from the dog coming into contact with the tissue samples.

Bone marrow aspirates were obtained with bone marrow aspiration needles (Argon Medical Devices^®^, Dallas, TX, USA) from the greater tubercle of the humerus, as described by Raskin & Messickin [42]. Hepatic and splenic biopsies were obtained by celiotomy and with the ligature fracture technique [43]. Finally, prescapular lymph node were biopsied with a biopsy punch (Premier^®^, Plymouth Meeting, PA, USA) as previously described [44]. Tissues samples were marked and frozen at − 20 °C for future extraction of DNA and PCR analysis.

Biopsies obtained from spleen, liver and lymph node had an average weight of 200 mg (range 150–210 mg). The amount of whole blood obtained was 1.5 ml and the bone marrow biopsy obtained 0.6 ml on average (range 0.4–0.7 ml)

### DNA extraction

For the blood samples, the extraction of genomic DNA from the cellular package of the dogs’ samples was performed using the UltraClean Blood DNA Isolation Kit (MoBio Lab®, Carlsbad, CA, USA), according to the manufacturer’s instructions.

The other tissues were handled in a sterile fashion prior to the extraction of DNA. For the extraction of DNA from the biopsies, the protocol was modified with the previous addition of lysis reagents [45]. The tissues were then macerated with the use of a low-velocity drill (Jorvet Lab^®^, Loveland, CO, USA) and a dental burn (JOTA Technical®, Rüthi, Switzerland). Once each tissue was macerated, DNA extraction was performed in the same way as for the blood.

### PCR amplification and analysis

Detection of *E. canis* DNA was achieved with the use of nested PCR molecular test. Initially, to amplify the *Ehrlichia* spp. *16S* rRNA gene, 2 pmol of primers ECC (5′′-AGA ACG AAC GCT GGC GGC CAA GC-3′) and ECB (5′-CGT ATT ACC GCG GCT GCT-3′) were used [28]. In the second PCR, to amplify the *E. canis 16S* rRNA gene, 2 pmol of primer HE-3(5′-TAT AGG TAC CGT CAT TAT CTT CCC TAT-3′) combined with the reverse primer ECA (5′-CAA TTA TTT ATA GCC TCT GGC TAT AGG AA-3′) were used [28, 46].

Initially, the PCR was performed in a thermocycler (Bio-Rad^®^ C-1000 Touch, Hercules, CA, USA) starting at 94 °C for 1 min followed by 35 cycles of 94 °C for 1 min (denaturation), 60 °C for 1 min (hybridisation) and 72 °C for 3 min (extension). This was followed by 94 °C for 5 min and then 40 cycles of 94 °C for 1 min (denaturation), 60 °C for 1 min (hybridisation), and 72 °C for 1 min (extension), as described previously [28, 46, 47].

### Statistical analyses

A multivariate logistic regression model was used for the response variable ‘infection’ which was binary (dummy variable) with y = 1 if positive, and y = 0 if negative, depending on two explanatory variables: blood positivity (two levels) and positivity in four separate tissues (four levels). Therefore, the model was: infection = blood + tissue + error.

The model analysed separately infection in both groups of dogs. In each group, the model compared infection among the four tissues using statistical tests ‘z’ between pairs of tissues, using a multiple-comparison Scheffe test.

Comparison of the proportions of positive and negative results in blood, lymph node, liver and spleen samples were performed using Chi square and Fisher’s exact tests with the FREQ procedure of SAS (9.0). Significance was considered with a *P*-value of < 0.05.

## Results

Of the 59 dogs analysed in this study, 28 (47.45%) showed a positive result for *E. canis* by PCR of blood samples, and 31 (52.55%) were negative. When evaluating the 28 dogs that were positive by PCR of blood samples, it was observed that 16 (57.14%) were also positive by PCR of some of the tissues. Otherwise, when analysing dogs with negative PCR results in blood (*n* = 31) and comparing them with the results of PCR in different tissues of the same dogs, it was observed that 19 dogs (61.30%) presented positive results for *E. canis* in some of the tissues and 12 (38.70%) were negative in all tissues biopsied.

The tissue biopsy with the highest number of positive samples was the bone marrow, with 26 (44.60%). Positive results from bone marrow samples occurred in both positive and negative blood samples. For example, 10 dogs (35.71%) that were positive by PCR of blood samples were also positive in PCR of the bone marrow (Table 1). Furthermore, it was found that 12 of 19 cases (63.15%) were positive with negative PCR of blood samples (Table 2). In half of the negative cases (*n* = 6), the results of the PCR of other tissues were negative. Conversely, in two cases, the PCR was positive for all tissues analysed.

**Table 1:** Comparison between positive blood-PCR of *E. canis* and the PCR results in other tissues

Blood PCR	Bone marrow PCR	Spleen PCR	Liver PCR	Lymph node PCR
+	+	+	+	–
+	+	+	+	–
+	+	+	+	+
+	+	+	+	+
+	–	+	+	–
+	+	+	+	–
+	+	+	+	–
+	–	+	+	–
+	+	+	+	–
+	+	+	+	–
+	–	+	+	–
+	–	+	–	–
+	–	–	–	–
+	–	+	–	–
+	+	–	–	–
+	+	–	–	–
+	+	+	–	–
+	–	–	–	–
+	+	–	–	–
+	–	–	–	–
+	–	–	–	–
+	–	+	–	–
+	–	–	–	+
+	+	+	+	–
+	–	–	–	–
+	–	–	–	–
+	–	–	–	–
+	–	–	–	–

*Key*: +, positive; –, negative

**Table 2:** Comparison between negative blood-PCR of *E. canis* and PCR results in other tissues

Blood PCR	Bone marrow PCR	Spleen PCR	Liver PCR	Lymph node PCR
–	–	+	+	–
–	–	+	+	–
–	–	–	–	–
–	–	+	+	–
–	+	+	+	+
–	+	–	+	+
–	–	–	–	–
–	+	+	+	–
–	+	–	–	–
–	–	–	–	–
–	–	+	–	–
–	+	+	+	–
–	+	+	+	+
–	–	–	–	–
–	–	–	–	–
–	–	–	–	–
–	–	+	–	–
–	–	–	–	–
–	–	–	–	–
–	+	–	–	–
–	+	–	+	–
–	−	–	–	–
–	+	–	–	–
–	–	–	–	–
–	+	–	–	–
–	–	–	–	–
–	+	–	–	–
–	–	–	–	–

*Key*: +, positive; –, negative

The tissue with the second highest number of positive results was the spleen, with a prevalence of 42.37% (*n* = 25). When analysing PCR-positive blood samples, 16 samples (57.14%) were also positive in PCR of spleen (Table 1). In blood PCR-negative dogs, the splenic tissue showed 9 (47.36%) positive PCR results, although there were spleen-only positive samples on two occasions (Table 2). Also, on two occasions the PCR was positive for all the tissues analysed. The remaining of the combinations are presented in Tables 1 and 2.

The liver had 22 PCR-positive cases (37.28%) from the total samples evaluated. Of the PCR-positive blood samples, 12 (42.85%) were also positive for the liver tissue (Table 1). Similarly, with the spleen, of the 19 PCR-negative blood samples, 10 (52.63%) were positive for the liver tissue. In the negative blood samples, there was one liver-only positive result (Table 2). In addition, the PCR was positive in all tissues twice. Finally, the tissue with the fewest positive results in the study was the lymph node, with 5 cases (8.47%). In the PCR-positive blood samples, only 2 cases were positive (10.52%; Table 1). On the other hand, the blood samples negative by PCR were positive for lymphatic tissue in 3 cases, representing 15.78%. In none of these three cases was the lymph node the only tissue with positive results (Table 2).

Considering infection in the four tissues, the infection rate was the same in both negative and positive dogs in PCR of blood samples (*P* > 0.05). The infection in tissues of negative dogs was an average rate of 0.23 ± 0.05, and for positive dogs was 0.35 ± 0.04 (*df* = 233, *P* < 0.001).

## Discussion

In the present study, of the 59 clinically healthy dogs analysed, 47.45% had a positive result for *E. canis* with PCR of blood samples. In addition, PCR recognised a higher prevalence of *E. canis* in different tissues of naturally infected dogs, in those with both positive and negative results by PCR of blood samples. With these results it was demonstrated that some dogs suspected of presenting subclinical ehrlichiosis, presented *E. canis* DNA in various tissues, even though they had negative PCR results from blood.

At the present time, diagnosis by PCR is more useful than serology for the differentiation of concurrent infections and co-infections with diverse *Ehrlichia* spp. and is used for treatment monitoring [46]. However, in naturally-occurring CME, the diagnostic sensitivity and optimal tissue for PCR testing in the untreated dog or in the post-treatment setting has not yet been clarified [46]. Results obtained at this point demonstrate that in dogs with naturally-occurring CME infection it is feasible to detect *E. canis* in different tissues, even if they have negative blood tests. Additionally, in the acute phase of infection, *E. canis* is easily detected in blood, while in the subclinical and chronic phases there is the possibility of false negatives. Therefore, some tissues are more appropriate for sampling, such as the bone marrow and the spleen [8, 9, 13, 48], an argument that has been corroborated by the present investigation. This study does not suggest performing tissue PCR for routine diagnosis of CME in dogs because performing biopsies in dogs with no clinical signs is impractical. However, sampling tissues may be relevant in understanding the distribution of CME in dogs.

Comparative information on the spread and presence of *E. canis* by PCR analysis in multiple organs is limited, especially in dogs with the natural form of the disease, although some research has been done in experimentally inoculated dogs. For example, it is proven that PCR is effective in detecting *E. canis* in diverse tissues of dogs with experimental disease [12]. In the same way, it has been described that the spleen is a tissue that can be useful to demonstrate the presence of *E. canis* DNA by PCR [8, 9]. In addition, the possibility of dogs in the subclinical phase being negative to PCR in blood samples and positive to PCR of splenic aspirates has also been established [8]. Splenic aspirates have previously been performed to detect *E. canis* DNA by PCR. Previous research has shown that dogs that were blood-positive were also positive to splenic aspirates, compared to those that were negative in blood [7]. These results differ from those obtained in the present investigation, where a prevalence of 42.37% (*n* = 25) was obtained. Furthermore, of the 19 blood PCR-negative dogs, nine (47.36%) were positive by PCR in the splenic biopsies.

It has been revealed that in the acute phase of disease, splenic aspirates are not superior to blood samples for detection of ehrlichial DNA by PCR. However, splenic aspirates are superior to blood in the evaluation of the response to therapy in experimentally treated dogs, because *E. canis* DNA could be detected in the spleen after its elimination from the blood [8].

The results of the present study also differ from previous reports in which the number of dogs positive and negative for *E. canis* by PCR is similar in blood samples and splenic aspirates. The results revealed that DNA of *E. canis* was isolated in 29 (72.5%) spleen samples and in 30 (75%) whole blood samples; and ehrlichial DNA was not isolated in 11 (27.5%) spleen samples and in 10 (25%) whole blood samples [10].

The difference between the other studies and the present investigation is the spleen tissue analysed. In our study, DNA was obtained through splenic biopsy, whereas in others DNA was obtained from blood through splenic aspirates. In another investigation, it was found that out of 78 dogs with splenic disease, only one was positive for *E. canis* by PCR in a splenic biopsy [49]. The present study creates the expectation of performing research to establish the most suitable technique to obtain *E. canis* DNA from the spleen in dogs by comparing splenic aspirates with biopsies, including those taken with minimally invasive techniques, such as ultrasound-guided or laparoscopic methods.

Furthermore, another important difference in our study is that the tissue with the highest number of positive samples was the bone marrow, in contrast to a previous report that obtained more positives from aspirates of the spleen [8]. Nevertheless, other studies have demonstrated that other tissues besides the spleen are better in detecting *E. canis* by PCR. For example, some authors describe results similar to those obtained in the present study and show that *E. canis* DNA was most often amplified from bone marrow [50, 51]. But, in these cases, there was experimental disease, and PCR was performed using aspirates. On the other hand, in one study on biopsies of dog cadavers, contrary to the results of the present study, none of the bone marrow biopsies was positive for *E. canis* by PCR [10].

An important limitation of the present study was the absence of blood analysis, especially blood counts. This could have established in a more accurate way the dogs presenting with the subclinical phase of monocytic ehrlichiosis [46]. However, it can be assumed that positive dogs were in this phase, since they were clinically healthy.

*Ehrlichia canis* is widespread throughout the different body systems of infected dogs. In addition, the molecular detection of *E. canis* DNA has shown that it can be present in different target organs [13, 52, 53]. In the subclinical and chronic phases, *E. canis* could be ‘hiding’ in splenic macrophages [8]. In this case, the spleen may be the principal reservoir of *E. canis*, probably because it has an abundance of macrophages. Moreover, some studies propose that it is the last organ to contain the microorganism before its elimination [8, 54]. Therefore, when containing a large number of bacteria, the spleen is considered by some authors as the organ of choice for molecular detection in different phases of the disease [4, 8, 48, 55]. Although in our study *E. canis* DNA was detected in the spleen, our results differ slightly from this statement, since it was the third most affected organ, surpassed by the bone marrow and liver. However, our results are similar to those of other studies that suggested that the spleen was inferior when compared to other tissues [10, 12, 49, 50].

## Conclusions

In conclusion, results of this study could be applicable in some cases where the diagnostic sensitivity of PCR may be suboptimal [46]. In some special cases, it will be necessary to search for *E. canis* DNA in different organs by molecular methods. In this study we have demonstrated that although infection in organs was 30% lower in dogs negative by PCR on blood samples, a considerable number of dogs (*n* = 19 or 61.30%) showing negative results by blood PCR were positive for *E. canis* in some organs. Dogs with positive blood results were positive in three tissues (liver, bone marrow and spleen) in 48% of cases. At the same time, these three tissues were more positive than the lymph node, which was positive in only 8% of the samples evaluated, and was four times lower than in any of the other three tissues. Dogs with negative results in blood showed 33% detection of *E. canis* DNA in the spleen, liver and bone marrow; however, the presence of DNA was higher in liver and bone marrow than in the lymph node. Because in some cases DNA was detected in only one of these tissues, it is proposed that biopsies be performed of at least these three. This assertion is stipulated for other rickettsial diseases, such as *Anaplasma* spp., where blood samples are routinely used for screening, but in persistently infected dogs with intermittent or low-level bacteraemia other tissues might be useful [56]. The results open the possibility of performing similar research aimed at detecting *E. canis* by PCR of different tissues in treated dogs that continue to show signs or alterations in blood tests, as well as in dogs that show signs suggestive of the disease but have negative results in serological and molecular blood analyses.

## Data Availability

The data supporting the conclusions of this article are included within the article and its additional files. Raw data used or analysed during the present study are available from the corresponding author upon reasonable request.

## Abbreviations

ACVIM: American College of Veterinary Internal Medicine
DNA: deoxyribonucleic acid
CME: canine monocytic ehrlichiosis
EDTA: ethylenediaminetetraacetic acid
ELISA: enzyme-linked immunosorbent assay
IFA: immunofluorescence antibody test
PCR: polymerase chain reaction
